# Outcomes of Frail Patients While Waiting for Kidney Transplantation: Differences between Physical Frailty Phenotype and FRAIL Scale

**DOI:** 10.3390/jcm11030672

**Published:** 2022-01-28

**Authors:** María José Pérez-Sáez, Dolores Redondo-Pachón, Carlos E. Arias-Cabrales, Anna Faura, Anna Bach, Anna Buxeda, Carla Burballa, Ernestina Junyent, Marta Crespo, Ester Marco, Leocadio Rodríguez-Mañas, Julio Pascual

**Affiliations:** 1 Nephrology Department, Hospital del Mar, 08003 Barcelona, Spain; doloredondop@gmail.com (D.R.-P.); cariascabrales@psmar.cat (C.E.A.-C.); afaura@psmar.cat (A.F.); abach@psmar.cat (A.B.); abuxeda@psmar.cat (A.B.); cburballa@psmar.cat (C.B.); ejunyent@psmar.cat (E.J.); mcrespo@psmar.cat (M.C.); julpascual@gmail.com (J.P.); 2 Physical Medicine & Rehabilitation Department, Parc de Salut Mar (Hospital del Mar-Hospital de L’ESPERANÇA), 08003 Barcelona, Spain; emarco@psmar.cat; 3 Rehabilitation Research Group, Hospital del Mar Research Institute, Universitat Autònoma de Barcelona, 08003 Barcelona, Spain; 4 Geriatrics Department, Hospital Universitario de Getafe, 28905 Madrid, Spain; leocadio.rodriguezs@salud.madrid.org

**Keywords:** frailty, FRAIL, kidney transplant waiting list

## Abstract

Frailty is associated with poorer outcomes among patients waiting for kidney transplantation (KT). Several different tools to measure frailty have been used; however, their predictive value is unknown. This is a prospective longitudinal study of 449 KT candidates evaluated for frailty by the Physical Frailty Phenotype (PFP) and the FRAIL scale. During the study period, 296 patients received a KT, while 153 remained listed. Patients who did not get receive a transplant were more frequently frail according to PFP (16.3 vs. 7.4%, *p* = 0.013). Robust patients had fewer hospital admissions during the 1st year after listing (20.8% if PFP = 0 vs. 43.4% if ≥1, and 27.1% if FRAIL = 0 vs. 48.9% if ≥1) and fewer cardiovascular events (than FRAIL ≥ 1) or major infectious events (than PFP ≥ 1). According to PFP, scoring 1 point had an impact on patient survival and chance of transplantation in the univariate analysis. The multivariable analysis corroborated the result, as candidates with PFP ≥ 3 had less likelihood of transplantation (HR 0.45 [0.26–0.77]). The FRAIL scale did not associate with any of these outcomes. In KT candidates, pre-frailty and frailty according to both the PFP and the FRAIL scale were associated with poorer results while listed. The PFP detected that frail patients were less likely to receive a KT, while the FRAIL scale did not.

## 1. Introduction

The prevalence of frailty among advanced chronic kidney disease (CKD) patients has been well established as high, and it is significantly higher in those who require hemodialysis (HD) as renal replacement therapy [1]. A wide variety of frailty scales have been used in population-based studies, and similar heterogeneity has been found in studies with renal patients [2]. The Physical Frailty Phenotype (PFP) defined by Fried et al. [3] is the most frequently applied among both dialysis and kidney transplant (KT) patients, although many others have also been utilized [2]. 

Differences among frailty scales are mainly due to the different aspects or domains that they take into consideration when evaluating a patient for frailty [2,4]. Hence, there are scales focused on patient comorbidities, cognition, social aspects, and objective measurements of individual physical reserve. This can result in different frailty phenotypes. Regardless of which domains the scales evaluate, i.e., which frailty phenotype are defining, health outcomes have been shown to be poorer in frail patients [2]. On the other hand, the time and resources consumed by each frailty tool are different. While some of them can be performed in less than one minute, others may take longer and require the use of devices and/or the involvement of health-care professionals for assessment [5].

The agreement between scales has been proven to be only fair in the best-case scenario [6,7], and population-based studies have targeted their efforts toward finding the best frailty metric adapted to each specific setting [4,5]. In pursuit of a disease-specific frailty metric, a few studies have evaluated which might be the best-known frailty tool to apply in the CKD population, with poor correlation with outcomes when comparing different frailty scales being reported thus far [8,9]. Potentially, depending on which frailty phenotype better fits with renal patients, one scale could have better sensitivity than another in predicting poor outcomes. On the other hand, depending on the individual clinical practice of each center, one scale might be more affordable to perform than others.

KT candidates present with worse health outcomes while listed [10,11,12] and have lower access to transplantation [13,14] depending on their frailty status. In addition, results after transplantation are influenced by patients’ baseline frailty status [15,16,17]. However, it is unknown which scale has the best correlation with these outcomes and, therefore, which would be the most suitable to apply in this specific population. 

Our aim in this study was to analyze different clinical outcomes among KT candidates while waiting for transplantation according to two different frailty scales: the PFP, which is the scale most used in the CKD population, and the FRAIL scale, which is less time consuming and more affordable to perform in clinical practice.

## 2. Materials and Methods

### 2.1. Study Design

This was a prospective cohort study of patients with advanced CKD at Hospital del Mar, Barcelona, Spain. Between June 2016 and June 2020, 449 patients were evaluated for frailty at the time they were referred for inclusion on the KT waiting list. During the study period, 153 patients remained on the KT waiting list and 296 received a KT. We grouped ‘waitlisted’ patients and included all KT candidates, regardless of their final status at the end of follow-up. Median time to follow-up was 26 months in those who did not get transplanted [interquartile range (IQR) 16–39] and 15 months (4–27) in those who received a transplant. Clinical and epidemiological variables were collected from our local database. 

### 2.2. Ethics

The Institutional Review Board of Hospital del Mar approved the study, and all enrolled participants provided written informed consent. The study followed the principles of the declaration of Helsinki, only relying on the official center database.

### 2.3. Frailty Assessment

Two different frailty assessment tools were used: PFP3 and the FRAIL scale [18]. PFP comprises five components: shrinking (self-report of unintentional weight loss of 4.5 Kg during the past year), weakness (grip strength below an established cut-off based on sex and body mass index (BMI)), exhaustion (self-report), low activity (kilocalories per week below an established cut-off) and slowed walking speed (walking time of 4.5 m below an established cut-off by sex and height). The FRAIL scale includes five questions (all of them self-reported) assessing fatigue, resistance, ambulation, illness, and loss of weight. In both scales, each component or question scores 0/1 depending on its presence or absence. Robust patients were defined as a score 0, pre-frail as those who ranked 1–2, and frail patients were defined by a score ≥ 3. In order to increase the power of the study, pre-frail and frail categories were joined for the analysis, as has previously been done in other cohorts [19,20].

### 2.4. Study Variables

For baseline characteristics, we included demographics (age, sex, ethnicity and BMI); social data (education as defined by four categories: none, primary education, secondary education, and tertiary education, family or social support as defined by its presence or absence, economic incomes as defined by three categories: non-regular incomes, retired with pension, and active worker with salary); and clinical data (comorbidities such as hypertension, diabetes mellitus, chronic cardiac and pulmonary diseases, type of renal replacement therapy, etc.). In addition, we assessed basic activities of daily living using the Barthel scale (disability if score ≤ 90) [21,22] and instrumental activities of daily living using the Lawton–Brody scale [23] (disability if <8 in women and <5 in men).

Regarding outcomes, we analyzed hospital admissions during the first year after listing (considered as >24 h admitted to a hospital and assessed in 277 patients who spent ≥ 12 months on the waiting list); major events while listed were as follows: cardiovascular events (stroke, heart failure episode, coronary ischemic event, peripheral vascular disease revascularization and/or amputation), major infectious episodes (including only those that required intravenous treatment with antibiotics), cancer onset, and dialysis access problems (including those related to the peritoneal catheter and those related to the vascular access for hemodialysis), and were analyzed only in those who remained on the KT waiting list (*n* = 153) along with chance of exclusion from the KT waiting list (only in those who remained on the KT waiting list (*n* = 153)), chance of transplantation (considering the whole cohort, *n* = 449), and patient mortality while listed (only in those who remained listed (*n* = 153)).

### 2.5. Statistics

Continuous variables were expressed as mean ± standard deviation (SD) or as median and IQR, according to normal distribution. Categorical data were expressed as absolute numbers and percentages. Comparisons of baseline characteristics between two groups were made using the Chi-square or Fisher’s exact test to analyze categorical variables, Student’s *t*-test for continuous variables with normal distribution, and Mann–Whitney test for non-parametric variables. ANOVA test was used to compare quantitative variables with normal distribution when three categories were present. Patient survival and chance of transplantation were estimated using Kaplan–Meier curves, applying the log-rank test. Univariate and multivariable Cox regressions were performed to estimate the hazard ratio (HR) and confidence intervals (CI) for patient survival or transplantation probability. The following variables were included in the univariate analysis: patient age, sex and comorbidity, dialysis vintage and frailty status according both PFP and FRAIL scale. These variables were chosen based on their clinical relevance. In the multivariable analysis, only those variables with a *p*-value < 0.1 were considered. Statistical analysis was performed using SPSS version 27 software (IBM, Armonk, NY, USA); *p*-values < 0.05 were considered statistically significant. 

## 3. Results

KT candidates presenting with at least one PFP criterion (pre-frail or frail patients) were 71.3%, while this percentage was lower when using FRAIL scale (45%). The number of patients presenting ≥ 3 criteria (frail patients) was 10.5% according to PFP and 3.6% according to FRAIL. Table 1 displays the whole cohort characterization and frailty criteria distribution according to PFP and the FRAIL scale.

### 3.1. Comparisons among Candidates Who Received a KT during the Study Period and Those Who Remained Non-Transplanted

Out of a total of 449 KT candidates, 296 received a KT during the study period while 153 remained listed (Table 1). The WL patients included 66 who were included in the KT WL at the end of follow-up, 23 patients who were excluded during the follow-up because of clinical issues (*n* = 18) or because they wanted to be (*n* = 5), 23 patients who were never waitlisted because of clinical problems detected during the transplant work-up, and 8 who were never waitlisted because they did not reach an estimated glomerular filtration rate (eGFR) below 15 mL/min. Patients who eventually received a transplant had better socioeconomic status than those who remained waitlisted (6.1 vs. 15% with non-regular incomes, respectively) and less frequently presented with heart failure (3.4 vs. 10.5%). Regarding PFP criteria distribution and total score, they presented with less slowness (10.8 vs. 19% of waitlisted patients), and fewer were frail (7.4 vs. 16.3%). On the contrary, although they scored better for resistance according to the FRAIL scale (6.1 vs. 15.7%), the FRAIL criteria distribution at the time of evaluation was similar between those who later received a KT and those who remained on the waiting list.

### 3.2. Clinical Outcomes of Frail Patients While Waiting for Transplantation

Two-hundred and seventy-seven candidates spent ≥12 months on the KT waiting list. 

Among robust patients, 20.8 and 27.1% required at least one hospital admission during their first year after evaluation according to the PFP and FRAIL scale, respectively (Table 2). Considering pre-frail and frail patients together, this percentage increased to 43.4% (PFP) and 48.9% (FRAIL). 

We analyzed clinical outcomes while listed in those who remained waitlisted (*n* = 153). Patients scoring ≥ 1 for PFP had more frequent major infectious events than robust ones (35.1% vs. 17.9%, respectively). Contrarily, FRAIL scale better discriminated between those patients who had a cardiovascular event while listed (49.3% if they scored ≥ 1 vs. 28% if they scored 0) and those who did not (Table 3). As a result of these events, the rate of waiting list exclusion was higher among those who were pre-frail or frail according to PFP (57 vs. 23.1% of robust patients) and according to the FRAIL scale (63.4 vs. 35.4%) (Table 3).

### 3.3. Mortality of Frail Patients While Waiting for Transplantation

Thirty-four patients had died at the end of follow-up. Of those, one patient was actively included in the KT waiting list, two were not included because their eGFR was not below 15 mL/min, five had never been included because of clinical issues, and 26 had been previously excluded due to clinical problems.

**Figure 1:**
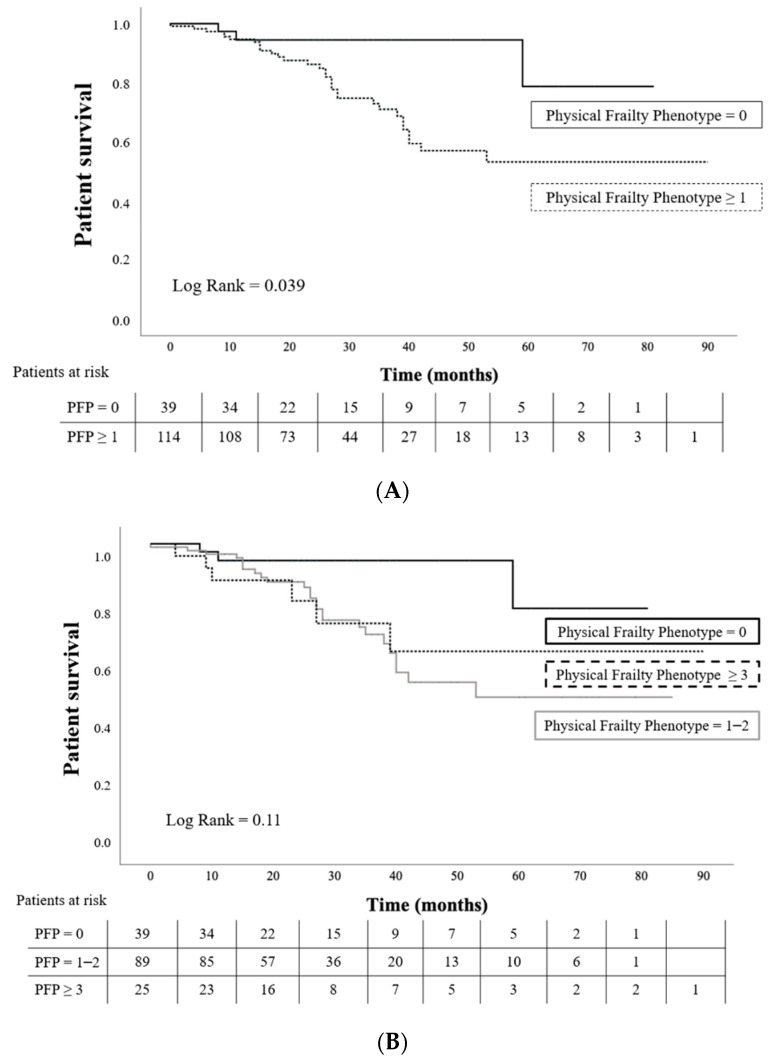

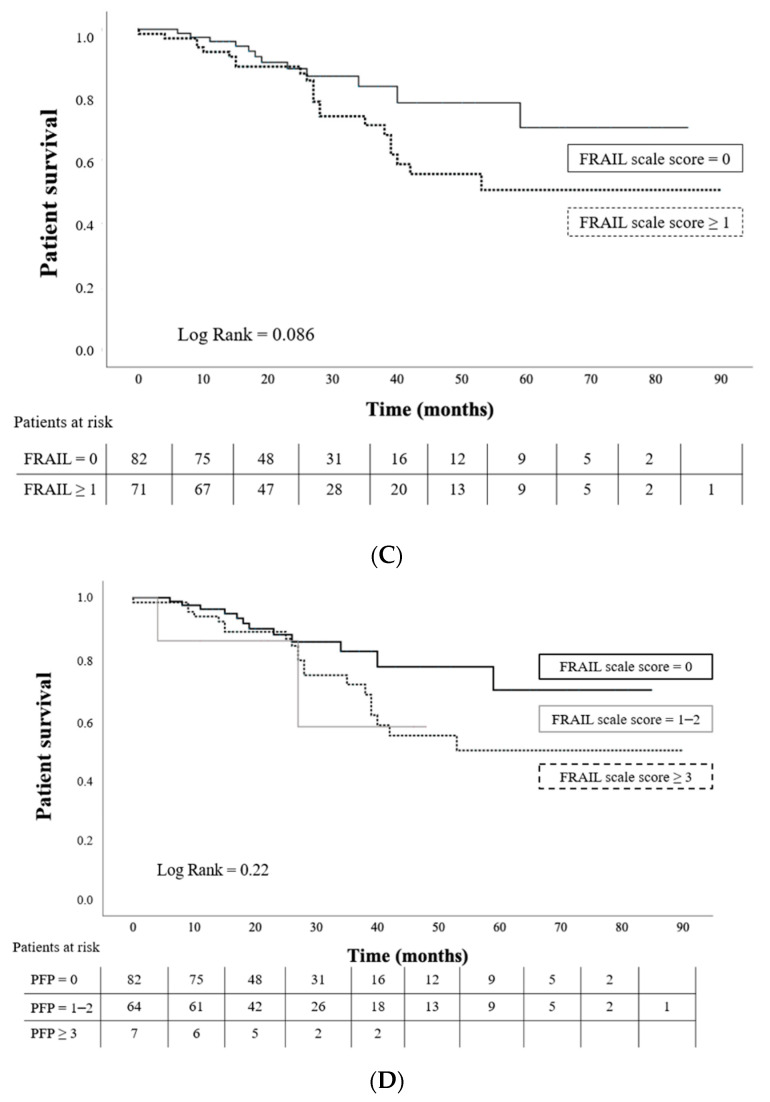
Kaplan–Meier curves of patient survival while listed according to their PFP and FRAIL scale score (*n* = 153). Time to follow-up (median IQR) 26 (16–39) months. (**A**) Patient survival among robust (PFP = 0) and both pre-frail and frail patients together (PFP ≥ 1); *p* = 0.039; (**B**) Patient survival among robust (PFP = 0), pre-frail (PFP = 1–2), and frail patients (PFP ≥ 3); *p* = 0.11; (**C**) Patient survival among robust (FRAIL = 0) and both pre-frail and frail patients together (FRAIL ≥ 1); *p* = 0.086; (**D**) Patient survival among robust (FRAIL = 0), pre-frail (FRAIL = 1–2), and frail patients (FRAIL ≥ 3); *p* = 0.22.

The univariate analysis of patient mortality while listed (*n* = 153) according to PFP score at the time of inclusion showed that robust patients had lower mortality (7.7 vs. 27.2% if PFP ≥ 1, *p* = 0.039, Figure 1A), although this difference lost its statistical significance when the three categories were considered (Figure 1B). In contrast, the FRAIL scale failed to detect this difference with both two and three categories (Figure 1C,D). When an adjusted multivariable Cox analysis was performed, only age (HR 1.098 per year) and cardiovascular disease (HR 3.43 if any cardiovascular disease was present) were associated with patient mortality. PFP ≥ 1 almost reached statistical significance for higher risk of mortality (HR 4.07, *p* = 0.09) (Table 4).

Twelve patients died of infection (five of them of COVID-19), ten patients of cardiovascular causes, eight of unknown causes (probably sudden death), and four of cancer.

### 3.4. Chance of Transplantation among KT Candidates and Its Relationship with Frailty Status

**Figure 2:**
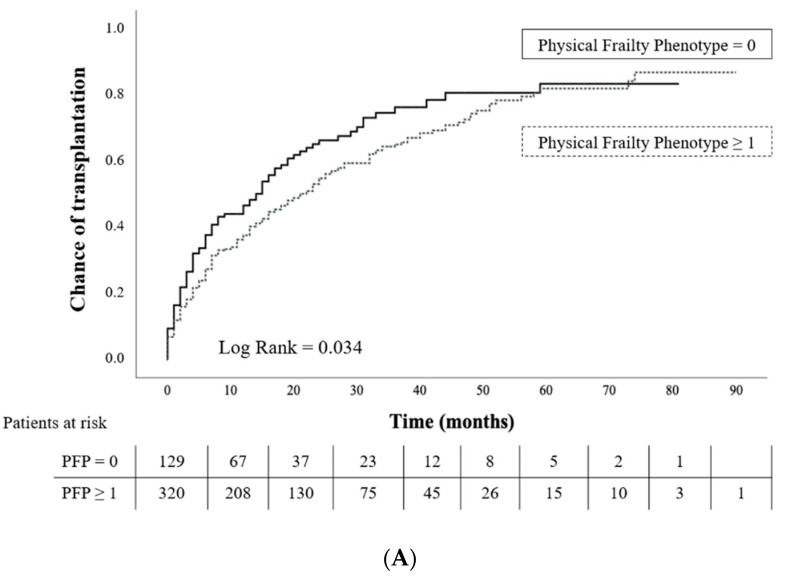

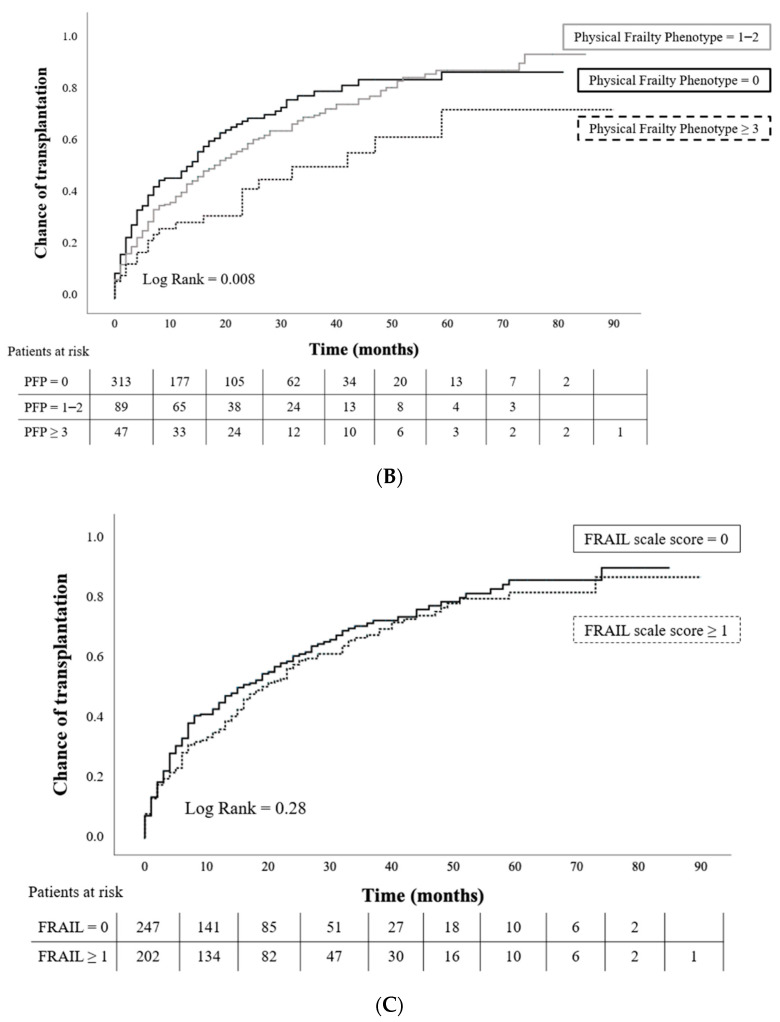

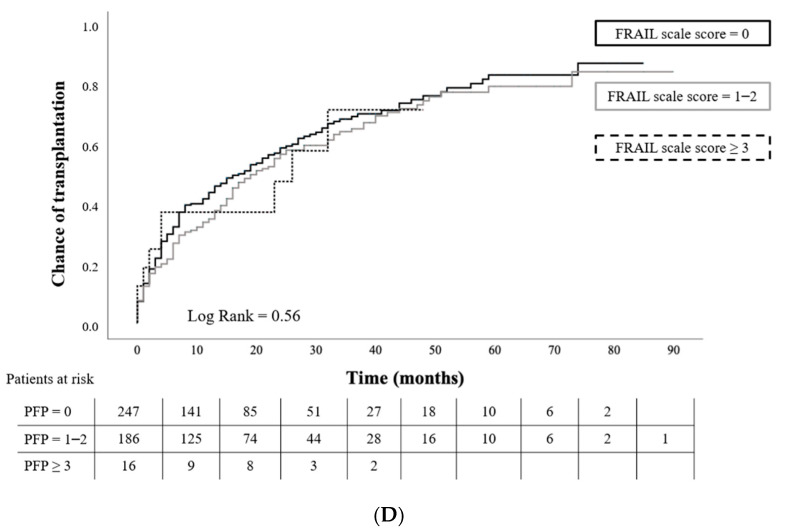
Kaplan–Meier curves of patient chance of transplantation while listed according to their PFP and FRAIL scale score (*n* = 449). Time to follow-up 15 (4–27) months. (**A**) Chance of transplantation among robust (PFP = 0) and both pre-frail and frail patients together (PFP ≥ 1); *p* = 0.034; (**B**) Chance of transplantation among robust (PFP = 0), pre-frail (PFP = 1–2), and frail patients (PFP ≥ 3); *p* = 0.008; (**C**) Chance of transplantation among robust (FRAIL = 0) and both pre-frail and frail patients together (FRAIL ≥ 1); *p*= 0.28; (**D**) Chance of transplantation among robust (FRAIL = 0), pre-frail (FRAIL = 1–2), and frail patients (FRAIL ≥ 3); *p* = 0.56.

Considering the whole cohort (*n* = 449), patients scoring ≥1 for PFP had a lower chance of obtaining a KT than those who were robust (Figure 2A), and this chance was gradually lower if more criteria were present (Figure 2B). However, the FRAIL scale was not associated with transplant probability (Figure 2C,D). In the multivariable analysis, cardiovascular disease was negatively associated with the chance of transplantation during follow-up (HR 0.72), while dialysis vintage was positively associated (HR 1.005 per month). PFP ≥ 3 candidates had a lower chance of receiving a transplant as well (HR 0.45) (Table 5).

## 4. Discussion

In this prospective study carried out in a cohort of 449 KT candidates, we analyzed frailty impact in terms of both clinical outcomes and patient survival while listed according to two different metrics (PFP and FRAIL scale) performed at the time of waiting list evaluation. We found higher rates of hospital admissions and major clinical events among those candidates who were pre-frail and frail according to both scales. Mortality was four-fold higher if patients scored ≥ 1 for PFP (*p* = 0.09), and if they scored ≥ 3 for PFP they had 55% less probability of transplantation while listed. The FRAIL scale was not associated with these outcomes.

Studies regarding KT candidates have established frailty as a major condition that impacts patient survival while listed [10,11,12,13]. Although many scales have been used both in KT candidates and recipients [2], PFP has the monopoly, probably because most of the research comes from the same group [10,11,13]. However, no studies have compared different scales and their potential association with outcomes.

In US cohorts, prevalence of frail KT candidates according to PFP scale has been established between 13.3 and 21% [10,11,13,17,24]. In our study, frail patients represented 10.5% (PFP) and 3.6% (FRAIL) of the whole cohort. In addition, differences were found in frailty prevalence among KT active candidates. Patients who finally receive a transplant presented with ≥3 Fried criteria in 7.4% of cases vs. 16.3% of those who remained listed. In contrast, the FRAIL scale revealed a lower percentage of frail patients, and without differences between those who were transplanted and those who were not. Clinicians must be aware of this difference in frailty prevalence between PFP and the FRAIL scale, which reaches 71.3% vs. 45% when pre-frail patients are considered (probably because PFP is more sensitive than FRAIL, as it involves objective measurements).

Hospital admission rate prior to KT is a strong determinant of waitlist mortality and transplant probability [25] and of poorer patient and graft survival after transplantation [25,26]. Frailty has been associated with hospitalizations in hemodialysis patients [27]. We described a higher rate of admissions during the first year after evaluating for transplantation in those KT candidates who were pre-frail and frail by both PFP and the FRAIL scale. Furthermore, PFP pre-frail and frail patients suffered more infectious events, while FRAIL pre-frail and frail patients suffered more cardiovascular events. Both scales were able to discriminate those candidates who were removed from the waiting list, even when only one point of score was present.

Thus far, it seems that both PFP and FRAIL scale would have the same predictive value for bad outcomes during the time the patient is waiting for KT. In larger US cohorts, frailty as determined by the PFP has shown an impact on patient mortality after listing, with an increased risk between 70 and 119% among those KT candidates who were frail [11,13]. In addition, a high comorbidity burden has been described as impacting patient survival only in robust candidates, as when frailty is present this comorbidity loses its significance [10]. We found that only frailty according to the PFP was associated with patient mortality in the univariate analysis (7.7% in robust patients and 27.2% in pre-frail or frail ones), although this significance was lost in the multivariable analysis (probably due to the small number of patients). Therefore, this difference might be due to other confounders such us comorbidity or disability, both of which are more frequent within frail patents. On the contrary, FRAIL failed to discriminate survival probability while listed. This could be related to a smaller number of patients being frail according to the FRAIL scale, or to the different frailty phenotype both scales are catching, as the FRAIL scale accounts for comorbidity and ignores physical reserve.

Likelihood of transplantation is lower when frailty is present. Haugen et al. described results regarding frailty according to the PFP and access to transplantation in KT active candidates. From a total of 4558 patients, 21% turned out to be frail, and they presented with 32% less probability to undergo a KT [13]. Chance of transplantation in our cohort was determined by PFP and FRAIL scale status. We found that frail patients according to the PFP had 55% less chance to be transplanted than robust patients. The FRAIL scale did not differentiate this outcome at the time of waiting list evaluation.

This study has the inherent limitations of a descriptive one-center study, thus, external validation may not be assumed. Frail patients accounted only for a small percentage of the sample, and the statistical power may be low. In addition, the degree of patient HLA sensitization might have played a role in the likelihood of transplantation; however, this information was not available. However, to our knowledge, this is the first study to compare the predictive value of bad outcomes of two different scales at the time of inclusion on the KT waiting list. Comparing scales may have importance, as they can be used depending on individual clinical practice and/or resources. PFP considers physical aspects that FRAIL does not. On the contrary, FRAIL considers comorbidity as part of frailty without measuring physical capacity. This might provide a lower percentage of frail patients (i.e., a less sensitive scale). However, it is unknown if these patients identified as frail by Fried and not by FRAIL have poorer outcomes. Although PFP appears to catch more CKD frail patients compared to the FRAIL scale (10.5 vs. 3.6%), being frail by both scales is related to poorer outcomes, and the FRAIL scale might be a good screening tool in cases where clinicians need to implement an easy-to-perform, faster option. 

## 5. Conclusions

Transplant units should incorporate frailty evaluation in patient assessment. This may be of crucial importance for proper patient information and clinician decisions, as well as for the detection of patients at risk and the implementation of interventional approaches to reverse frailty before transplantation. The decision about which scale to use at the time the candidate is evaluated will depend on many factors, as its feasibility is different in terms of time and resources consumed. Frailty status according to the PFP scale provides more accurate insight than the FRAIL scale about KT waiting list patient prognosis.

## Figures and Tables

**Table 1:** Baseline characteristics and frailty characterization of the total cohort at the time of evaluation for the KT WL (*n* = 449). Comparisons were made between patients who remained as KT candidates (*n* = 153), and those who received a KT during the study period (*n* = 296).

	All (*n* = 449)	WL ^1^ (*n* = 153)	KT (*n* = 296)	*p*-Value ^a^
Sociodemographics				
Age (years, mean ± sd)	60.4 ± 14.1	61.5 ± 13.5	59.8 ± 14.3	0.20
Sex (female, *n* (%))	141 (31.4)	50 (23.7)	91 (30.7)	0.67
Ethnicity (Caucasian, *n* (%))	420 (93.5)	141 (92.2)	279 (94.3)	0.21
BMI (Kg/m^2^, mean ± sd)	27.9 ± 5.2	28.5 ± 5.2	27.7 ± 5.1	0.17
Education (no/primary, *n* (%))	282 (62.8)	99 (64.7)	183 (61.8)	0.54
Deficient family support, *n* (%)	64 (14.2)	19 (12.4)	45 (15.3)	0.41
Socioeconomic status (non-regular incomes, *n* (%))	41 (9.1)	23 (15)	18 (6.1)	0.002
Comorbidities				
Hypertension, *n* (%)	432 (96.2)	148 (96.7)	284 (96.3)	0.80
Diabetes mellitus, *n* (%)	164 (36.5)	63 (41.2)	101 (34.2)	0.15
Heart failure, *n* (%)	26 (5.8)	16 (10.5)	10 (3.4)	0.002
Ischemic coronary disease, *n* (%)	73 (16.3)	32 (20.9)	41 (13.9)	0.055
Peripheral vasculopathy, *n* (%)	41 (9.1)	16 (10.5)	25 (8.4)	0.48
Cerebral vasculopathy, *n* (%)	35 (7.8)	13 (8.5)	22 (7.4)	0.69
Chronic obstructive pulmonary disease, *n* (%)	34 (7.6)	15 (9.8)	19 (6.4)	0.19
RRT modality, *n* (%)				
Hemodialysis	295 (65.7)	107 (69.9)	188 (63.5)	
Peritoneal dialysis	93 (20.7)	22 (14.4)	71 (24)	0.06
Low clearance	61 (13.6)	24 (17.5)	37 (12.5)	
Dialysis vintage (months, median [IQR])	7 (0–14)	6 (−2–13.5)	7 (1–14)	0.19
Disabilities				
Disability for activities of daily living *, *n* (%)	35 (8.7)	16 (12.3)	19 (7)	0.077
Disability for instrumental activities of daily living ^#^, *n* (%)	88 (22)	33 (26)	55 (20.1)	0.19
Physical Frailty Phenotype				
Shrinking, *n* (%)	77 (17.1)	27 (17.6)	50 (16.9)	0.84
Exhaustion, *n* (%)	108 (24.1)	43 (28.1)	65 (22)	0.14
Low physical activity, *n* (%)	25 (5.6)	12 (7.8)	13 (4.4)	0.13
Slowness, *n* (%)	61 (13.6)	29 (19)	32 (10.8)	0.02
Weakness, *n* (%)	243 (54.1)	90 (58.8)	153 (51.7)	0.15
Hand grip (Kg, mean ± sd)	26.8 (9.4)	26.3 (9.8)	27.1 (9.2)	0.30
PFP 3 categories (0, 1–2, and ≥3)				
0	129 (28.7)	39 (25.5)	90 (30.4)	
1–2	273 (60.8)	89 (58.2)	184 (62.2)	0.013
≥3	47 (10.5)	25 (16.3)	22 (7.4)	
PFP 2 categories (0 and ≥1)				
0	129 (28.7)	39 (25.5)	90 (30.4)	0.27
≥1	320 (71.3)	114 (74.5)	206 (69.6)	
FRAIL scale				
Fatigue, *n* (%)	108 (24.1)	43 (28.1)	65 (22)	0.14
Resistance, *n* (%)	42 (9.4)	24 (15.7)	18 (6.1)	<0.001
Ambulation, *n* (%)	20 (4.5)	10 (6.5)	10 (3.4)	0.12
Illness, *n* (%)	55 (12.2)	18 (11.8)	37 (12.5)	0.82
Loss of weight, *n* (%)	86 (19.2)	31 (20.3)	55 (18.6)	0.66
FRAIL 3 categories (0, 1–2, and ≥3)				
0	247 (55)	82 (53.6)	165 (55.7)	
1–2	186 (41.4)	64 (41.8)	122 (41.2)	0.68
≥3	16 (3.6)	7 (4.6)	9 (3)	
FRAIL 2 categories (0 and ≥1)				
0	247 (55)	82 (53.6)	165 (55.7)	0.66
≥1	202 (45)	71 (46.4)	131 (44.3)	

KT, kidney transplant; WL, waiting list; BMI, body mass index; RRT, renal replacement therapy; IQR, interquartile range; PFP, Physical Frailty Phenotype. ^1^ WL patients included 66 patients who were included in the KT WL at the end of follow-up, 23 patients who were excluded during the follow-up because of clinical issues (*n* = 18) or because they wanted to be (*n* = 5), 23 patients who were never waitlisted because of clinical problems detected during the transplant work-up, and 8 who were never waitlisted because they did not reach an eGFR below 15 mL/min. * Barthel ≤ 90; ^#^ Lawton-Brody < 8 if women and <5 if men; ^a^ Comparisons were made between WL and KT categories.

**Table 2:** Hospitalizations while first year on the KT WL according to PFP and FRAIL scale (*n* = 277).

PFP			
	0 (*n* = 72)	≥1 (*n* = 205)	*p*-Value
Hospitalization requirement during 1st year on the WL, *n* (%)	15 (20.8)	89 (43.4)	<0.001
Number of hospitalizations, *n* (%)			0.09
0	56 (77.8)	115 (56.1)	
1	9 (12.5)	56 (27.3)	
2	2 (2.8)	22 (10.7)	
3	3 (4.2)	9 (4.4)	
>3	2 (2.8)	3 (3)	
**FRAIL Scale**			
	**0** **(*n* = 144)**	**≥1** **(*n* = 133)**	***p*-Value**
Hospitalization requirement during 1st year on the WL, *n* (%)	39 (27.1)	65 (48.9)	<0.001
Number of hospitalizations, *n* (%)			<0.001
0	104 (72.2)	67 (50.4)	
1	29 (20.1)	36 (27.1)	
2	4 (2.8)	20 (15)	
3	4 (2.8)	8 (6)	
>3	3 (2.1)	2 (1.5)	

KT, kidney transplant; WL, waiting list; PFP, Physical Frailty Phenotype.

**Table 3:** Clinical outcomes while listed according to PFP and FRAIL scale (*n* = 153).

PFP			
	0 (*n* = 39)	≥1 (*n* = 114)	*p*-Value
Cardiovascular event, *n* (%)	10 (25.6)	48 (42.1)	0.07
Major infection event, *n* (%)	7 (17.9)	40 (35.1)	0.04
Neoplasia, *n* (%)	2 (5.1)	13 (11.4)	0.25
Dialysis access problem, *n* (%)	4 (10.3)	22 (19.3)	0.19
Any event, *n* (%)	10 (25.6)	59 (51.9)	0.005
Number of events > 1, *n* (%)	5 (12.8)	29 (25.4)	0.10
WL exclusion during the follow-up, *n* (%)	9 (23.1)	65 (57)	<0.001
**FRAIL scale**			
	**0** **(*n* = 82)**	**≥1** **(*n* = 71)**	***p*-Value**
Cardiovascular event, *n* (%)	23 (28)	35 (49.3)	0.007
Major infection event, *n* (%)	22 (26.8)	25 (35.2)	0.26
Neoplasia, *n* (%)	8 (9.8)	7 (9.9)	0.98
Dialysis access problem, *n* (%)	9 (11)	17 (23.9)	0.033
Any event, *n* (%)	31 (37.8)	38 (53.1)	0.05
Number of events > 1, *n* (%)	13 (15.9)	21 (29.6)	0.03
WL exclusion during the follow-up, *n* (%)	29 (35.4)	45 (63.4)	<0.001

PFP, Physical Frailty Phenotype; WL, waiting list. Any event includes: cardiovascular event, major infection event, neoplasia and dialysis access problem.

**Table 4:** Univariate and multivariable analysis of patient death while listed (*n* = 153). Time to follow-up 26 (16–39) months.

	Univariate			Multivariable		
	HR	CI 95%	*p*-Value	HR	CI 95%	*p*-Value
Age (per year)	1.008	1.003–1.013	0.03	1.098	1.003–1.015	0.003
Sex (ref: male)	0.637	0.309–1.316	0.22			
Race (ref: Caucasian)	3.30	1.13–9.61	0.03	1.35		0.19
Hypertension	20.99	0.02–228.17	0.51			
Diabetes mellitus	1.32	0.67–2.60	0.43			
Any cardiovascular disease	2.37	1.20–4.68	0.01	3.43	1.35–8.66	0.009
Chronic obstructive pulmonary disease	1.57	0.61–4.06	0.35			
Hemodialysis as RRT modality (ref: HD)	0.72	0.27–1.87	0.50			
Dialysis vintage (per month)	1.004	0.99–1.01	0.43			
PFP (ref: 0)						
≥1	3.24	0.99–10.61	0.05	4.07	0.78–21.15	0.09
PFP (ref: 0)						
1–2	2.87	0.71–11.4	0.13	4.05	0.77–21.19	0.09
≥3	3.35	1.01–11.10	0.04	4.37	0.64–29.14	0.13
FRAIL scale (ref: 0)						
≥1	1.83	0.90–3.70	0.09	1.51	0.62–3.70	0.35
FRAIL scale (ref: 0)						
1–2	1.81	0.88–3.70	0.15	1.51	0.66–3.47	0.32
≥3	2.06	0.46–9.27	0.43	1.95	0.38–9.98	0.41

HR, hazard ratio; CI, confidence interval; RRT, renal replacement therapy; HD, hemodialysis; PFP, Physical Frailty Phenotype. Four multivariable analyses were performed, including (1) PFP ≥ 1; (2) PFP 1–2 and ≥ 3; (3) FRAIL scale ≥ 1; and (4) FRAIL scale 1–2 and ≥3. Age and any cardiovascular disease remained significant in all four models.

**Table 5:** Univariate and multivariable analysis of chance of the transplantation while listed (*n* = 449). Time to follow-up 15 (4–27) months.

	Univariate			Multivariable		
	HR	CI 95%	*p*-Value	HR	CI 95%	*p*-Value
Age (per year)	0.99	0.98–1.01	0.64			
Sex (ref: male)	0.80	0.62–1.02	0.08	0.81	0.62–1.06	0.14
Hypertension	1.01	0.55–1.84	0.97			
Diabetes mellitus	0.83	0.65–1.06	0.14			
Any cardiovascular disease	0.72	0.55–0.94	0.01	0.72	0.54–0.95	0.02
Chronic obstructive pulmonary disease	0.82	0.51–1.30	0.40			
Hemodialysis as RRT modality (ref: HD)	0.94	0.63–1.41	0.78			
Dialysis vintage (per month)	1.005	1.001–1.008	0.01	1.005	1.001–1.009	0.007
PFP (ref: 0)						
≥1	0.76	0.60–0.98	0.03	0.78	0.59–1.03	0.08
PFP (ref: 0)						
1–2	0.82	0.64–1.06	0.13	0.83	0.63–1.09	0.19
≥3	0.49	0.30–0.78	0.003	0.45	0.26–0.77	0.004
FRAIL scale (ref: 0)						
≥1	0.88	0.70–1.11	0.29	0.84	0.65–1.08	0.19
FRAIL scale (ref: 0)						
1–2	0.88	0.70–1.11	0.30	0.85	0.66–1.10	0.23
≥3	0.87	0.44–0.71	0.70	0.70	0.32–1.51	0.37

HR, hazard ratio; CI, confidence interval; RRT, renal replacement therapy; HD, hemodialysis; PFP, Physical Frailty Phenotype. 4 multivariable analyses were performed, including: (1) PFP ≥ 1; (2) PFP 1–2 and ≥ 3; (3) FRAIL scale ≥ 1; and (4) FRAIL scale 1–2 and ≥3. Any cardiovascular disease and dialysis vintage both remained significant in all four models.

## Data Availability

The data presented in this study are available on request from the corresponding author. The data are not publicly available due to ethical and privacy reasons.
